# Molecular detection of multiple viral targets in untreated urban sewage from Greece

**DOI:** 10.1186/1743-422X-8-195

**Published:** 2011-04-27

**Authors:** Petros A Kokkinos, Panos G Ziros, Αggeliki Mpalasopoulou, Alexis Galanis, Apostolos Vantarakis

**Affiliations:** 1 Environmental Microbiology Unit, Department of Public Health, School of Medicine, University of Patras, Rion, GR 26504, Greece; 2 Department of Molecular Biology and Genetics, Democritus University of Thrace, Alexandroupolis, GR 68100, Greece

## Abstract

**Background:**

Urban sewage virological analysis may produce important information about the strains that cause clinical and subclinical infections in the population, thus supporting epidemiological studies.

**Methods:**

In the present study, a twenty one-month survey (November 2007 to July 2009) was conducted in order to evaluate the presence of human adenoviruses (hAdV), hepatitis A viruses (HAV), hepatitis E viruses (HEV), Noroviruses (NoV), and human Polyomaviruses (hPyV) in untreated sewage samples collected from the inlet of Patras' municipal biological wastewater treatment plant, located in southwestern Greece. Nucleic acid amplification techniques were applied for viral nucleic acid detection. Positive samples were confirmed by sequencing and comparative phylogenetic analysis was performed on the isolated viral strains.

**Results:**

In total, viruses were detected in 87.5% (42/48) of sewage samples. AdVs, PyVs, HAV, and NoVs were detected in 45.8% (22/48), 68.8% (33/48), 8.3% (4/48), and 6.3% (3/48) of the samples collected from the plant's inlet, while HEV was not detected at all. Adenovirus types 8 (Ad8), 40 (Ad40) and 41 (Ad41) were recognized, while JC and BK polyomaviruses were recorded. Noroviruses were identified as GII.4. HAV was typed as genotype IA.

**Conclusions:**

Our study demonstrates the advantages of environmental surveillance as a tool to elucidate the molecular epidemiology of community circulating viruses. We underline the need of environmental surveillance programs in countries such as Greece with inadequate and problematic epidemiological surveillance system and no environmental surveillance system currently in action.

## Background

Wastewater presents a timely dynamic collection point where many physical, chemical, and biological substances of our society are brought to a central location. Any type of infection within a community is likely to lead to pathogen excretion in bodily fluids/substances and therefore, transported into the community sewage system. A wide variety of pathogenic organisms pass through municipal wastewater treatment systems, including viruses [1].

The enteric viruses found in human stool and urine belong to more than 140 types of which adenovirus (AdV), hepatitis A virus (HAV), norovirus (NoV) genotype I and II, rotavirus (RV), enterovirus (EV), and polyomavirus (PyV) are those most often detected in the environment [2-7]. HAdVs are associated with sporadic cases and occasional outbreaks of gastroenteritis [7]. Hepatitis A represents worldwide around 50% of the total hepatitis cases and hepatitis A virus has been linked to several waterborne outbreaks. Hepatitis E is less frequent than hepatitis A, and in industrialised countries is thought to be spread zoonotically, principally from swine [8]. NoVs are an important cause of epidemic acute gastroenteritis, and waterborne outbreaks of NoV associated gastroenteritis are well documented [9]. The human polyomaviruses have been shown to be present in high concentrations in the sewage, and their specificity as human viruses may be useful as a marker for faecal pollution of anthropogenic origin [10].

Bibliography has been enriched the last few years by several studies which have demonstrated the advantage of environmental surveillance as an additional tool to determine the epidemiology of different viruses circulating in a given community [8,11-16]. The availability of improved detection techniques, combined with an increased awareness of gastroenteritis-causing viral pathogens, has also led to the establishment of surveillance systems in various countries, since other enteric viruses responsible for gastroenteritis and hepatitis have replaced enteroviruses as the main target for detection [8,17]. Environmental surveillance can provide valuable supplementary information, particularly in urban populations with absent or questionable surveillance, when persistent virus circulation is suspected or frequent virus re-introduction is perceived [18]. In Greece, there is no specific national surveillance system for gastroenteric viruses, and this explains the limited available data on enteropathogenic viruses.

In the present study, a twenty one-month survey (November 2007-July 2009) was conducted in order to evaluate the presence of human adenoviruses (hAdV), hepatitis A viruses (HAV), hepatitis E viruses (HEV), Noroviruses (NoV), and human Polyomaviruses (hPyV) in sewage samples collected from the inlet of a municipal biological wastewater treatment plant, located in southwestern Greece. The study aimed to enrich the poor data on environmental virological studies in Greece, to demonstrate the benefit of environmental surveillance as a tool, to determine the epidemiology of viruses circulating in a given community, and to underline the need for the design and support of similar long-term studies in our country.

## Methods

### Wastewater treatment plant and sampling

The municipal wastewater treatment plant of the present study receives urban sewage from the city of Patras. The municipality has 171.616 inhabitants (census of 2001) and is located in south western Greece. The plant is officially registered as a secondary treatment plant with anaerobic digestion of the sludge. It treats 38.000 m^3 ^of urban sewage per day. The wastewater effluents are discharged into the Patraikos Gulf. From November 2007 to July 2009, forty eight (48) samples of untreated sewage were collected from the municipal sewage treatment plant. Samples were collected early in the morning, the first day of each sampling week. The samples were transferred to the laboratory into a cool box and they were immediately subjected to virological analysis for the detection of human AdVs, NoVs, HEV, HAV, and hPyVs.

### Sample concentration, viral extraction and biomolecular analysis

Sewage samples were processed as described elsewhere [7,19,20]. Viral nucleic acids were extracted from concentrated samples using the QIAamp Viral RNA mini-kit (Qiagen) according to the manufacturer's instructions. Reverse transcription polymerase chain reaction (RT-PCR) and nested PCR techniques have been used for the detection of human AdVs, HAV, HEV, NoVs, human PyVs, according to previously published protocols [19-21].

### Sequence analysis

Positive PCR products were purified using the QIAquick PCR purification kit (Qiagen, USA) and confirmed by sequencing (Sequencing unit, School of Medicine, University of Thessaly, Greece). The obtained nucleotide sequences were analyzed by BLAST program at the NIH website (NCBI, National Centre for Technology Control, NIH, USA), and were compared with each other and with other published sequences. Multiple alignments were performed with the Clustal X program. The neighbour-joining method has been applied for the phylogenetic tree analysis, the reliability of which was assessed by bootstrap resampling (1,000 pseudoreplicates), using MEGA 4.0.2 program.

## Results

### Virus Detection and Phylogenetic Analysis

In total, viruses were detected in 87.5% (42/48) of sewage samples. AdVs, PyVs, HAV, and NoVs were detected in 45.8% (22/48), 68.8% (33/48), 8.3% (4/48), and 6.3% (3/48) of the samples collected from the plant's inlet, while HEV was not detected at any sample.

**Figure 1:**
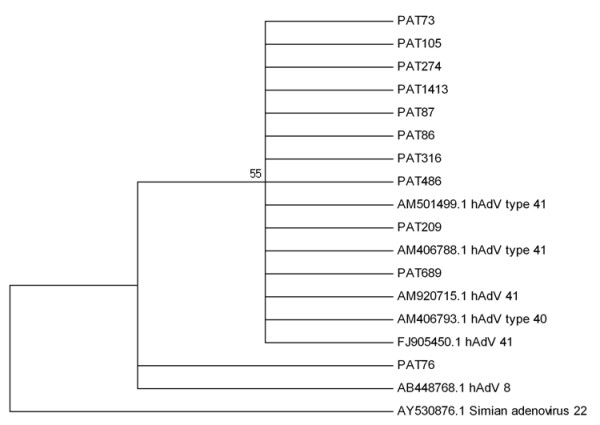
**Phylogenetic analysis of hAdV strains**. An NJ phylogenetic tree was constructed to represent phylogenetic relationships among eighteen hAdV strains. Eleven strains (abbreviated as PAT73, PAT105, PAT274, PAT1413, PAT87, PAT86, PAT316, PAT486, PAT209, PAT689, PAT76) were isolated from Patras' sewage samples. Seven reference strains the sequences of which were retrieved from GenBank database were included to the analysis. Reference strains belong to hAdV genotype 8, 40, and 41. Simian AdV 22 strain [GenBank: AY530876] was used as an out-group. The bootstrap confidence levels were obtained for 1,000 replicates.

**Figure 2:**
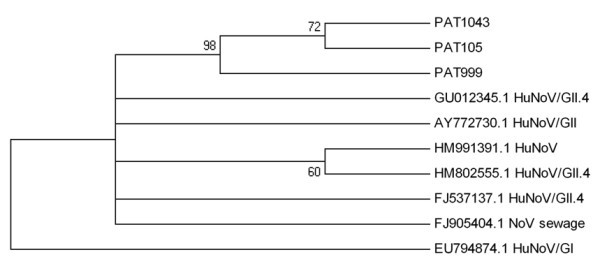
**Phylogenetic tree of NoV strains**. An NJ phylogenetic tree was constructed to represent phylogenetic relationships among three NoV strains of the present study (PAT1043, PAT105, PAT999) along with seven NoV reference strains. Numbers under branches are bootstrap percentage values, calculated from 1,000 bootstrap replicates. GenBank accession numbers and genotype of the reference sequences are included in the phylogram. Reference strains under the following accession numbers [GenBank: GU012345, AY772730, HM991391, HM802555, FJ537137, FJ905404, and EU794874], derived from Brazil, Germany, China, Hong Kong, USA, Tunisia and Belgium, respectively. The environmental reference NoV strain from Tunisy was isolated from treated sewage.

**Figure 3:**
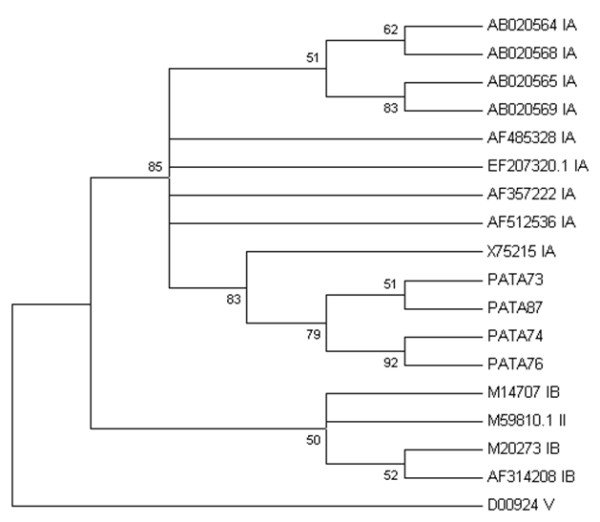
**Phylogenetic tree constructed to represent phylogenetic relationships among eighteen HAV strains**. Four HAV strains abbreviated as PAT73, PAT74, PAT76, PAT87 were isolated from Patras' sewage samples. Sixteen HAV references sequences were used for the phylogenetic tree constutcion. Nine reference strains corresponded to genotype IA [GenBank: AB020564, AB020568, AB020565, AB020569, EF207320, AF357222, AF512536, X75215], three to genotype IB [GenBank: M14707, M20273, AF314208], and one to genotype II [GenBank: M59810]. The bootstrap confidence levels obtained for 1,000 replicates are shown in the phylogram. Simian HAV strain [GenBank: D00924 genotype V] was used as an out-group.

**Figure 4:**
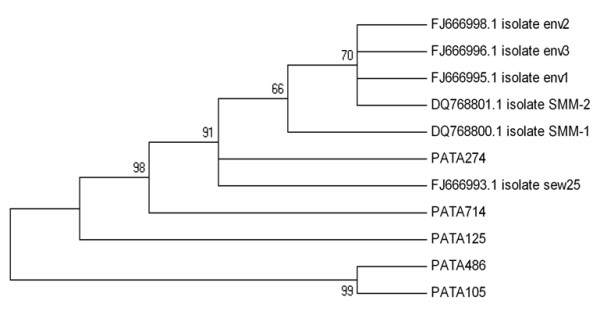
**Phylogenetic tree depicting the relationship between the environmental BK PyV strains of the present study compared with publicly available environmental BK sequences**. Numbers under branches are bootstrap percentage values, calculated from 1,000 bootstrap replicates. GenBank accession numbers of the reference sequences are included in the phylogram. Strains abbreviated as PAT105, PAT125, PAT274, PAT486, PAT714 were isolated from raw sewage samples collected from Patras' biological treatment plant. Isolates env1, env2 and env3 were isolated from environmental water contaminated with sewage, while isolates SMM-1, SMM-2, and sew25 derived from raw sewage samples.

**Figure 5:**
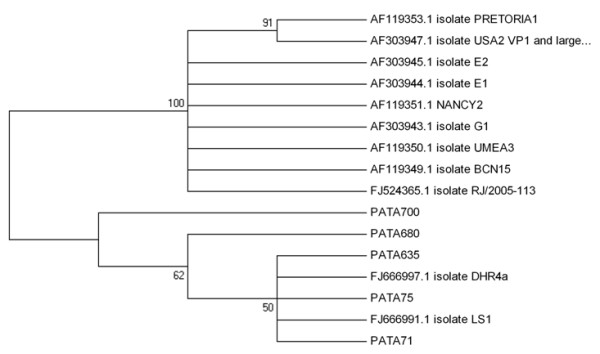
**Phylogenetic tree depicting the relationship between the environmental JC PyV strains of the present study compared with publicly available environmental JC sequences**. Nucleotide sequences of five Greek JC PyV strains isolated from raw sewage samples collected from the sewage treatment plant of the city of Patras (PAT, 71, PAT75, PAT635, PAT680, PAT700) were compared with fifteen reference environmental strains. GenBank accession numbers of the reference sequences are included in the phylogram. Reference strains abbreviated as PRETORIA1, USA2, E2, NANCY2, E1, G1, UMEA3, BCN15, DHR4a, RJ/2005-113, and LS1 were isolated from urban sewage derived from South Africa, United States, Egypt, Russia, Egypt, Greece, Czech Republic, United Kingdom, USA, Brazil, and USA respectively. Numbers under branches are bootstrap percentage values, calculated from 1,000 bootstrap replicates.

The phylogenetic analyses performed are summarized in Figures 1,2,3,4,5. Figure 1 shows the phylogenetic tree which was constructed to represent phylogenetic relationships among hAdV strains. Adenovirus types 8 (Ad8), 40 (Ad40) and 41 (Ad41) were recognized. Ten out of eleven hAdV strains isolated during the present study from Patras' sewage samples (abbreviated as PAT) cluster with reference strains types 40 and 41, while strain PAT76 clusters with the reference strain type 8 (AB448768). Simian AdV type 22 (AY530876) was used as an out-group for the analysis and forms a distinct clade to all human strains, as expected.

Figure 2 shows the NJ phylogenetic tree of the three Norovirus strains isolated during the present study along with seven NoV reference strains. Patras' NoV strains cluster with six clinical and one environmental NoV strain, all of genotype GII.4. The environmental reference NoV strain included to the analysis derived from Tunisia and was isolated from treated sewage. Norovirus strain (EU794874) of genotype GI forms a distinct clade to all other GII.4 strains of the study.

The phylogenetic relationships among four HAV strains isolated from Patras' sewage treatment plant and thirteen reference strains are presented in Figure 3. All Patras' HAV strains cluster with HAV reference strains of genotype IA. Simian HAV strain (D00924 genotype V) was used as an out-group for the analysis and forms a distinct clade to all environmental and clinical human strains, as expected.

Regarding the hPyVs, JC and BK polyomaviruses were recorded (Figures 4 &5). Phylogenetic trees depicting the relationship between the environmental BK and JC PyV strains of the present study compared with publicly available environmental BK and JC sequences are shown in figures 4 and 5, respectively. BK reference isolates were isolated from raw sewage and environmental water contaminated with sewage. The five Patras' JC PyV strains form a cluster with reference strains DHR4a, and LS1.

## Discussion

Monitoring of human pathogens in sewage is possible because they may be excreted in a range of bodily fluids, skin, and hair during active infection. The analysis of centralized wastewater allows detection of intentional, natural, or accidental contamination events. A person with an enteric viral infection may excrete as many as 10^14 ^viral particles per day and over 10^15 ^during the course of an infection [1]. During the last years, more attention has been focused on the sewage virological quality, the risk of virus-associated waterborne illness, the need for routine monitoring viral contamination and the environmental surveillance through the analysis of sewage [3,22,23].

To enrich the poor existing virological data, a twenty one-month survey (November 2007-July 2009) was conducted to examine the HAV, hAdVs, HEV, NoVs, and hPyVs presence in sewage samples collected from a biological wastewater treatment plant, located at the city of Patras.

Limited data on enteropathogenic viruses are available in Greece, because there is no specific surveillance system for viral gastroenteritis. The role of enteric viruses as a cause of gastroenteritis in Northwestern Greece was investigated by a 6-year study of stool samples from children hospitalized for acute gastroenteritis. Rotaviruses, noroviruses, adenoviruses and astroviruses were detected in 21.35%, 4%, 3.5% and 2.35%, respectively. Although enteric adenovirus types 40 and 41 predominated, non-enteric subgenera (A and C) were also causally implicated [24]. In the present study, the analysis of sewage samples mainly revealed adenoviruses types 40, and 41 and in one case a strain type 8 was identified (Figure 1). All NoV strains were typified as NoV GII.4 (Figure 2). GII.4 is the predominant genotype worldwide [25].

Hepatitis A virus appears in the stool of infected individuals 2-3 weeks before clinical illness [1]. According to the latest national cross-sectional seroprevalence survey of hepatitis A among Greek children, hepatitis A is intermediate endemic in Greece, and the National Advisory Committee for Immunization has included the hepatitis A vaccine in the GNIP from January 2008 [26]. In the present study, HAV was detected in four sewage samples and typified as genotype IA (Figure 3). In a previous study, one of five sewage samples from Patras tested positive for HAV [27]. Moreover, in one of our recent studies to characterize hepatitis A virus isolates from environmental and clinical samples in Greece, a 100% prevalence of subgenotype IA was recorded [28].

HEV is considered an emerging pathogen in industrialized countries. In the last years, some HEV strains associated with sporadic acute hepatitis have been isolated from human serum samples in European countries (i.e., Italy, Greece, Spain, and the United Kingdom) [27]. In a study to investigate the level of infection in regions where HEV is considered nonendemic by analyzing the excreted virus in the urban sewage of diverse geographic areas, HEV RNA was not detected in any of the four samples from Patras, Greece [27]. A molecular HEV screening performed on raw sewage samples from different wastewater treatment plants yielded positives at 16%, evenly distributed throughout Italy [29]. A study aimed to determine the prevalence of anti-HEV among haemodialysis patients of a semi-rural region in central Greece showed that the prevalence of anti-HEV was greater than in healthy blood donors [30]. In the present study, none of the analyzed sewage samples was found positive for HEV.

JCV and BKV Polyomaviruses are excreted in the urine and are spread in high concentrations in sewage. In addition to the potential use of polyomaviruses as a useful marker for pollution of anthropogenic origin, being able to detect and study these viruses in sewage provides an opportunity to evaluate and monitor those strains that are prevalent in specific geographical areas [10]. Polyomavirus BK infection in Greek renal transplant recipients has been reported by Zavos [31]. To test the involvement of BK virus (BKV), and JC virus (JCV), in prostate tumorigenesis, the prevalence of these viruses was tested in a series of human prostatic malignancies. The overall prevalence of polyomaviruses was 19% in the prostate cancer cases, and sequencing analysis revealed the presence of BKV in all samples [32]. In another study, to detect JC virus in a series of colon neoplasms from Greek patients, JCV sequence was detected in 61% of colon adenocarcinomas and in 60% of adenomas, at a viral load of 9 × 10^3 ^up to 20 × 10^3 ^copies/μg DNA [33]. The detection of JC virus DNA sequences and the expression of the viral regulatory protein T-antigen in tumors of the central nervous system from patients from Greece, USA and UK were described by Del Valle [34]. The characteristics of JCV excreted in the environment have been studied by analyzing sewage samples from divergent geographical areas. The phylogenetic analysis of JCV strains detected in the sewage of Barcelona (Spain), Umeå (Sweden), Nancy (France), Pretoria (South Africa), Patras (Greece), Cairo (Egypt), Washington, D.C. (USA), and diverse areas of Northern India showed their relationships with JCV strains previously described in urine or clinical samples in these geographic areas [35]. In our study, PyVs (JC and BK) have been detected in 68.8% of the analyzed samples. Phylogenetic trees depicting the relationship between the environmental BK and JC PyV strains of the present study compared with publicly available environmental (sewage or water contaminated by sewage) BK and JC sequences are presented in figures 4 and 5, respectively.

Sewage surveillance system has been shown to be more sensitive than reporting of clinical cases of serious illness in a community [1]. Data from the occurrence of viruses in raw sewage may provide an overview of the epidemiology of virus infections circulating in the community, and at the same time, reveal the occurrence of asymptomatic infections [8,27].

Our study enriches the poor available data on sewage virological quality, and demonstrates the advantages of environmental surveillance as a tool to elucidate the molecular epidemiology of community circulating viruses. We underline the need of environmental surveillance programs in countries such us Greece with limited epidemiological surveillance systems for viral gastroenteritis and no environmental surveillance system currently in action and we propose that similar long-term studies might be useful and offer a valuable and complementary tool to epidemiological surveillance systems.

## Competing interests

The authors declare that they have no competing interests.

## Authors' contributions

PK carried out the sequence alignments, constructed the phylogenetic trees and wrote the manuscript. PZ participated in the molecular analyses for HAV and HEV detection and helped to draft the manuscript. AM carried out part of the molecular analyses for viral detection in sewage samples. AG contributed in the molecular analyses for PyV detection. AV was responsible for setting up and coordinating the study, and drafted the manuscript. All authors read and approved the final manuscript.
